# New Insights on the Role of *N*^6^-Methyladenosine RNA Methylation in the Physiology and Pathology of the Nervous System

**DOI:** 10.3389/fmolb.2020.555372

**Published:** 2020-09-02

**Authors:** Georgia Dermentzaki, Francesco Lotti

**Affiliations:** ^1^Center for Motor Neuron Biology and Disease, Department of Pathology and Cell Biology, Columbia University, New York City, NY, United States; ^2^Department of Neurology, Columbia University, New York City, NY, United States

**Keywords:** RNA modifications, *N*^6^-methyladenosine, m^6^A, RNA metabolism, neurodevelopment, mood disorders, neurodevelopmental disorders, neurodegenerative disorders

## Abstract

RNA modifications termed epitranscriptomics represent an additional layer of gene regulation similar to epigenetic mechanisms operating on DNA. The dynamic nature and the increasing number of RNA modifications offer new opportunities for a rapid fine-tuning of gene expression in response to specific environmental cues. In cooperation with a diverse and versatile set of effector proteins that “recognize” them, these RNA modifications have the ability to mediate and control diverse fundamental cellular functions, such as pre-mRNA splicing, nuclear export, stability, and translation. *N*^6^-methyladenosine (m^6^A) is the most abundant of these RNA modifications, particularly in the nervous system, where recent studies have highlighted it as an important post-transcriptional regulator of physiological functions from development to synaptic plasticity, learning and memory. Here we review recent findings surrounding the role of m^6^A modification in regulating physiological responses of the mammalian nervous system and we discuss its emerging role in pathological conditions such as neuropsychiatric and neurodegenerative disorders.

## Introduction

Epitranscriptomics or “RNA epigenetics” refers to post-transcriptional RNA modifications that mediate several cellular processes necessary for maintaining RNA homeostasis. To date approximately 170 distinct modifications have been identified in coding and non-coding RNA (Boccaletto et al., 2018), with the most prominent and best characterized being *N*^6^-methyladenosine (m^6^A) methylation. m^6^A, the deposition of a methyl group on the adenosine base at the nitrogen-6 position (Bokar et al., 1997), was first discovered in 1974 (Desrosiers et al., 1974; Perry et al., 1975). However, it has only recently garnered increased attention due to advances in transcriptome-wide mapping methods that provide a greater resolution of its distribution on m^6^A-modified transcripts (Dominissini et al., 2012; Meyer et al., 2012; Linder et al., 2015) and the identification of enzymes that could reverse this modification (Jia et al., 2011; Zheng et al., 2013).

m^6^A is the most abundant internal RNA modification in mammalian mRNA with approximately 25% of mammalian mRNAs being modified, with an average of 1–3 modifications per transcript. It is preferentially installed near stop codons and 5′ and 3′ UTRs, as well as in introns and long internal exons but to a lesser extent, with a high degree of conservation between human and mouse (Dominissini et al., 2012; Meyer et al., 2012). m^6^A is installed by a methyltransferase complex termed the writer complex and removed by demethylases termed erasers. Effector proteins termed readers recognize m^6^A and mediate multifaceted functions of mRNA metabolism including splicing, nuclear export, stability, translation, microRNA processing, and subcellular targeting of m^6^A-modified mRNAs (Meyer and Jaffrey, 2017; Shi et al., 2019; Zaccara et al., 2019). To date a plethora of studies have implicated m^6^A in diverse biological functions including stem cell proliferation and differentiation (Heck and Wilusz, 2019), circadian rhythm (Hastings, 2013), spermatogenesis (Lin and Tong, 2019), hematopoiesis (Weng et al., 2019), immunoregulation (Zhang et al., 2019), metabolism (Zhong et al., 2020), and cancer (Huang et al., 2020).

Proper development of the nervous system is a highly coordinated, multi-step process that requires tight regulation to ensure proper function. As a consequence, deficits in neurodevelopment have been implicated in several brain disorders (Kiser et al., 2015; Meredith, 2015; Cardoso et al., 2019). Interestingly, m^6^A is highly enriched in the mammalian brain and is developmentally regulated with its expression peaking during adulthood (Meyer et al., 2012; Nainar et al., 2016; Chang et al., 2017). Several studies have highlighted the functional significance of m^6^A regulation in the physiology of the nervous system both during early development and in adulthood (Widagdo and Anggono, 2018; Li et al., 2019; Livneh et al., 2020). However, the role of m^6^A in maladapted responses of the nervous system is still poorly understood and has been the focus of recent studies that implicate m^6^A in neurodevelopmental, neuropsychiatric, and neurodegenerative disorders (Engel and Chen, 2018; Shafik et al., 2020). In this review, we will summarize the current body of knowledge surrounding the m^6^A effectors, the regulatory mechanisms that they govern, as well as their biological consequences in both physiology and pathology of the mammalian nervous system.

## m^6^A Machinery

**FIGURE 1:**
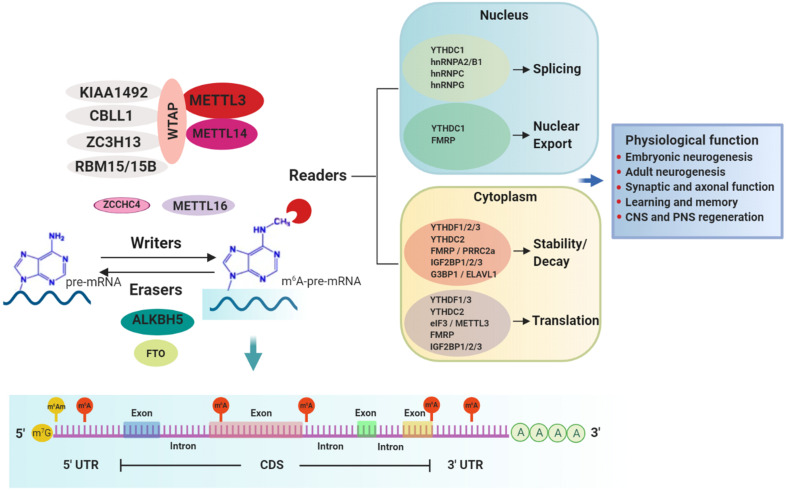
m^6^A-mediated mRNA regulation and physiological responses in the nervous system. m^6^A methylation in mRNA substrates is performed by writers. The best-characterized writer is a multi-subunit complex consisting of the core enzymes METTL3 (catalytic enzyme) and METTL14 and other regulatory proteins such as WTAP, KIAA1492, ZC3H13, RBM15/15B, and CBLL1. Other less characterized writer proteins are METTL16 and the recently discovered ZCCHC4. Two erasers, FTO and ALKBH5, mediate the reverse process of demethylation. m^6^A is preferentially installed near stop codons and 5′ and 3′ UTRs, as well as in introns (near 5′ and 3′ splice sites) and long internal exons but to a lesser extent. Conversely, m^6^Am, a modification regulated also by FTO, occurs only adjacent to the m^7^G (N7-methylguanosine) cap. m^6^A-modified mRNAs are recognized by a diverse group of RNA binding proteins called readers which regulate several aspects of the mRNA metabolism including splicing, nuclear export, decay, stability and translation, through which different physiological processes in the nervous system are accomplished.

m^6^A is installed co-transcriptionally in nascent transcripts by the writer complex. The core elements of this complex consist of methyltransferase-like 3 (METTL3), the catalytic component, and methyltransferase-like 14 (METTL14), which activates METTL3’s enzymatic activity and facilitates RNA substrate binding (Figure 1) (Schwartz et al., 2014; Wang X. et al., 2014; Liu K. et al., 2016; Sledz and Jinek, 2016; Wang et al., 2016). Additional regulatory subunits contribute to the activity and specificity of the writer complex. Wilms tumor 1-associating protein (WTAP) is essential for optimal substrate recruitment and METTL3/14 localization to the nuclear speckles (Zhong et al., 2008; Ping et al., 2014; Schwartz et al., 2014). KIAA1429 or vir-like m^6^A methyltransferase associated (VIRMA) mediates preferential m^6^A-deposition in the 3’ UTR and near stop codons (Yue et al., 2018). Cbl-proto-oncogene-like protein 1 (CBLL1, also known as HAKAI) is required for m^6^A deposition on mRNA-targets (Ruzicka et al., 2017). Zinc finger CCCH-type containing 13 (ZC3H13) is required for nuclear localization of the writer complex (Wen et al., 2018). RNA binding motif protein 15/15B (RBM15/15B) directs m^6^A-deposition in specific sites of mRNA substrates (Patil et al., 2016).

While METTL3/14 writer complex typically installs m^6^A in a sequence motif of DRACH (D = A, G or U; R = A or G; H = A,C or U) (Liu et al., 2014), another m^6^A writer, methyltransferase-like 16 (METTL16), places m^6^A in a sequence context of UAC(m^6^A)GAGAA on top of a hairpin RNA structure in selected mRNA targets, including MAT2A (Pendleton et al., 2017; Doxtader et al., 2018; Mendel et al., 2018). More recently, a new m^6^A writer, ZCCHC4, was identified to methylate primarily the 28S rRNA and also to interact with a subset of mRNAs (Ma et al., 2019). The proteins comprising the m^6^A-writer complex are mostly nuclear (Jia et al., 2011; Ping et al., 2014). However, several studies have reported these proteins can also be located in the cytoplasm where they mediate m^6^A deposition in mature mRNAs as well as being involved in methylation-independent functions (Alarcon et al., 2015b; Chen et al., 2015; Lin et al., 2016).

m^6^A can be reversed by erasers, of which only two have been identified to date, fat mass and obesity-associated protein (FTO) and AlkB homolog 5 (ALKBH5) (Figure 1). FTO, the first RNA demethylase identified, was reported to remove the methyl group of m^6^A in mammalian mRNA (Jia et al., 2011; Fu et al., 2013). However, further characterization of substrate spectrum of FTO showed that its demethylase activity is not only selective toward internal m^6^A in mRNA but also toward m^1^A in specific tRNAs, cap-m^6^Am in mRNA as well as m^6^Am in some snRNAs (Wei et al., 2018). Therefore, it is important to consider that FTO’s contribution to physiological and disease phenotypes that are discussed below should not be interpreted solely in conjunction with its function as m^6^A eraser. Conversely, ALKBH5 preferentially demethylates m^6^A in a consensus DRACH motif-dependent manner in specific transcripts and has no other known substrates (Zheng et al., 2013; Xu et al., 2014; Zhang et al., 2016, 2017; Tang et al., 2018). The expression patterns of ALKBH5 and FTO are distinct among tissues. ALKBH5 is highly expressed in testis and is essential for spermatogenesis (Zheng et al., 2013), whereas FTO, despite being ubiquitously expressed, is enriched in the brain, specifically in neurons (Gerken et al., 2007; McTaggart et al., 2011; Aas et al., 2017), implicating a key regulatory role in the nervous system.

## Regulation of mRNA Metabolism Through m^6^A Modification

m^6^A-dependent post-transcriptional gene regulation is mediated by RNA binding proteins (RBPs), termed readers, that recognize and decode the m^6^A epimark (Figure 1 and Table 1). Characterization of m^6^A readers has revealed the presence of different classes of RBPs, divergent binding modes and multifaceted downstream biological functions (Dominissini et al., 2012; Edupuganti et al., 2017). Direct RNA binding relies on a dedicated domain within the reader protein that recognizes the modification within a sequence motif. For example, proteins containing the YT521-B homology (YTH) domain, including cytoplasmic YTH domain family 1–3 (YTHDF1–3) and nuclear YTH domain containing 1–2 (YTHDC1–2) in mammals directly bind methylated transcripts to exert their effects on gene expression (Zhang et al., 2010). A different group of readers utilizes common RNA binding domains to bind m^6^A-containing RNAs, conferring greater access to RBPs through methylation-induced remodeling of local RNA structures (Liu et al., 2015). Several proteins fall into this category, including the heterogeneous nuclear ribonucleoproteins (hnRNPs) (Alarcon et al., 2015a; Liu et al., 2015; Liu N. et al., 2017; Wu et al., 2018), the fragile X mental retardation protein (FMRP) (Edupuganti et al., 2017; Zhang Z. et al., 2018), the insulin-like growth factor 2 mRNA-binding proteins 1–3 (IGF2BP1-3) (Huang et al., 2020) and the proline rich coiled-coil 2A (PRRC2A) protein (Wu et al., 2019). Not only does m^6^A attract RBPs, but recent studies have also identified RBPs that are instead repelled by the presence of m^6^A, such as Ras GTPase-activating protein-binding protein 1 (G3BP1) and G3BP2 proteins (Arguello et al., 2017; Edupuganti et al., 2017), adding another layer of complexity to m^6^A-dependent gene expression regulation.

**TABLE 1:** m^6^A readers involved in the physiology and pathology of the nervous system.

Reader	Function^1^	Binding	Physiology	Pathology	References^2^
YTHDC1	Splicing; nuclear export	Direct	Not determined	Cerebellar ataxia	Fernandez-Funez et al., 2000
YTHDC2	Stability; translation	Direct	Not determined	Autism spectrum disorder	Liu X. et al., 2016
YTHDF1	Translation	Direct	Learning and memory, synaptic function, axonal guidance, regeneration in the PNS	Not determined	Merkurjev et al., 2018; Weng et al., 2018; Shi et al., 2019; Zhuang et al., 2019
YTHDF2	Decay	Direct	Cortical neurogenesis	Not determined	Li et al., 2018
YTHDF3	Translation; decay	Direct	Synaptic function	Not determined	Merkurjev et al., 2018
HNRNPA2/B1	Pre-mRNA splicing; miRNA processing; decay	Indirect (RNA switch)	Not determined	ALS	Kim et al., 2013
FMRP	Nuclear export; stability; translation	Possibly direct	Neural differentiation, localization and function of dendritic RNAs	Fragile X syndrome; intellectual disability	Dictenberg et al., 2008; Kao et al., 2010; Chang et al., 2017; Edens et al., 2019
PRRC2A	Stability	Possibly direct	Specification and myelination of oligodendrocytes	Locomotive and cognitive defects	Wu et al., 2019

*^1^Function here refers to the role that m^6^A readers have in the different aspects of RNA metabolism. ^2^References cited for FMRP and PRRC2A refer to both Physiology and Pathology of the nervous system.*

### mRNA Splicing/Nuclear Export

The fact that m^6^A is present within exons (Dominissini et al., 2012) and further associated with pre-mRNA splicing factors (SRSFs) (Zhao et al., 2014; Xiao et al., 2016) strengthened the view that it may regulate splicing. YTHDC1 was shown to recruit SRSF3 to promote exon inclusion in an m^6^A-dependent manner, while blocking SRSF10, which favors exon skipping (Xiao et al., 2016). Several hnRNPs, which are known to control different aspects of mRNA metabolism, such as hnRNPC, hnRNPG, and hnRNPA2-B1 have also been described to interact with m^6^A-modified mRNAs through multiple proteomic screenings (Edupuganti et al., 2017; Liu N. et al., 2017). A study by Liu et al. (2015) described the presence of an “m^6^A-switch” mechanism where m^6^A alters the local structure in mRNA and long non-coding RNA to facilitate the binding of hnRNPC and promote alternative splicing of target mRNAs. hnRNPA2-B1 was also shown to bind the m^6^A-consensus motif *in vitro* and *in vivo* and was suggested to mediate, in part, the effects of m^6^A/METTL3 alternative splicing as well as microRNA processing (Alarcon et al., 2015a).

Apart from its m^6^A-mediated splicing role, YTHDC1 can interact with the SRSF3 to facilitate the nuclear RNA export factor 1 (NXF1)-mediated nuclear export of m^6^A-modified mRNAs (Roundtree et al., 2017). Toward the same direction, FMRP, an m^6^A reader with a yet undetermined binding mode, can also facilitate the nuclear export of m^6^A-modified mRNAs through the nuclear export protein CRM1 during neural differentiation (Edens et al., 2019).

### mRNA Stability/Decay

A possible link between m^6^A and mRNA stability was first suggested in the late 1970’s (Sommer et al., 1978), a finding that was further confirmed by later studies in which proteins of the writer complex (METTL3 and WTAP) were disrupted in both human and mouse cells (Batista et al., 2014; Liu et al., 2014; Schwartz et al., 2014). Thus far, the best characterized decay-inducing reader is YTHDF2. YTHDF2 can directly mediate mRNA decay either by binding to m^6^A-containing mRNAs and targeting them to P-bodies (Wang X. et al., 2014; Ries et al., 2019), or by recruiting the CCR4-NOT deadenylase complex to promote m^6^A-mRNA degradation independently from P-bodies (Du et al., 2016). YTHDC2, which can be both nuclear and cytoplasmic, has a dual role during early spermatogenesis, where it both enhances the translation efficiency of its targets while decreasing their mRNA abundance (Hsu et al., 2017). An additional route for YTHDF2-dependent mRNA decay has been recently reported, where m^6^A-containing RNAs are subjected to endoribonucleolytic cleavage via HRSP12 (adaptor protein) and RNase P/MRP (endoribonucleases) (Park et al., 2019). YTHDF2 was also suggested to act synergistically with YTHDF3, which might control the mRNA binding specificity, to promote m^6^A-mediated mRNA decay (Shi et al., 2017). However, the view that YTHDF proteins bind to specific m^6^A sites on selected mRNAs has been challenged by a new study proposing a different model to explain how YTHDF proteins influence the fate of m^6^A-modified mRNAs (Zaccara and Jaffrey, 2020). According to the authors, all three YTHDF proteins bind to all m^6^A site in a similar manner and they work in concert to promote mRNA degradation (Zaccara and Jaffrey, 2020).

While YTHDF proteins have been proposed to promote mRNA degradation, several indirect readers have been reported to enhance mRNA stability, such as FMRP, which likely competes with YTHDF2 for binding to m^6^A-containing mRNAs and the stress granule component G3BP1, which is repelled by m^6^A (Edupuganti et al., 2017; Zhang F. et al., 2018). IGF2BP1-3 were also demonstrated to stabilize m^6^A-containing mRNAs and enhance translation (Huang et al., 2018). In addition, a recent report added PRRC2A to the list of m^6^A readers (Wu et al., 2019), showing that recombinant PRRC2A prefers to bind a methylated probe and further stabilizing a critical m^6^A modified transcript required for myelination. Lastly, the stabilizing/destabilizing effect of m^6^A may also be promoted by human antigen R (HuR; AKA ELAV-like protein 1, ELAVL1), depending on the position of m^6^A and HuR-binding site in target mRNAs (Wang Y. et al., 2014).

### mRNA Translation

Methylation at either the 5′ UTR or 3′ UTR near the stop codon of mRNAs has been reported to promote translation in mammalian cell systems. Two readers of the cytoplasmic YTH-domain family, YTHDF1 and YTHDF3, were reported to mediate cap-dependent translation, presumably via 3′ UTR methylation of target transcripts in mammalian cells, by recruiting translation initiation factors (Wang et al., 2015; Li et al., 2017; Shi et al., 2018). Although both YTHDF1 and YTHDF3 can functionally associate with ribosomes, only YTHDF1 can directly interact with the translation initiation factor complex 3 (eIF3) (Li et al., 2017; Shi et al., 2018). However, a recent study challenged the prevailing model that YTHDF1 and YTHDF3 function as mRNA translation enhancers by proposing that all three YTHDF proteins act redundantly to promote m^6^A-mediated mRNA degradation (Zaccara and Jaffrey, 2020).

Irrespective of the controversial role of YTHDF1 and YTHDF3 on mRNA translation, m^6^A at the 5′ UTR of mRNAs can directly interact with the eIF3 and promote cap-independent translation, especially under stress-response conditions where the cap-dependent translation is repressed (Meyer et al., 2015; Zhou et al., 2015). Interestingly, METTL3 itself has also been suggested to act in the cytoplasm as an m^6^A reader and promote translation cooperatively with eIF3 in human cancer cells (Lin et al., 2016). Translational control by m^6^A has also been suggested via its interaction with the FMRP protein in HeLa cells (Arguello et al., 2017; Edupuganti et al., 2017). Further supporting m^6^A’s role in regulating translation, a recent study introduced a mechanism where m^6^A methylation in coding sequences promotes translational elongation by reducing ribosomal pausing via recruitment of the YTHDC2 reader (Mao et al., 2019).

### Spatial Regulation

RNA modifications, and m^6^A in particular, have been shown to influence the spatial distribution of their target mRNAs to exert their subcellular-specific functions. There is currently limited knowledge regarding how this process is being mediated through m^6^A and which proteins are involved in the guidance of the m^6^A-modified mRNAs in their final subcellular destination. The first evidence of m^6^A being involved in the spatial regulation of its mRNA targets came from a study by Zheng et al. (2013) where the demethylation activity of ALKBH5 significantly altered mRNA nuclear export. In this study ALKBH5-deficient cells showed increased cytoplasmic mRNA levels due to accelerated export in contrast to control cells where mRNAs were mainly accumulated in the nucleus, suggesting that m^6^A might be required for mRNA export.

Studies have linked cytoplasmic m^6^A with specific structures such as P-bodies and stress granules (SGs) (Gao et al., 2019; Ries et al., 2019; Fu and Zhuang, 2020) which constitute assemblies of mRNAs and RBPs involved in mRNA turnover or translation (Decker and Parker, 2012). m^6^A-modified mRNAs can interact with P-bodies via the YTHDF2 reader to promote their decay (Wang X. et al., 2014; Ries et al., 2019). Notably, Ries et al. (2019) demonstrated that YTHDF proteins can undergo liquid–liquid phase separation *in vitro* and in cells, and that this process is markedly enhanced by the presence of poly-methylated m^6^A-mRNAs rather than single m^6^A-mRNAs. These m^6^A-mRNAs/YTHDF complexes can then partition into different endogenous phase-separated compartments, such as P-bodies, SGs or neuronal RNA granules (Ries et al., 2019). Along the same line, another study showed that poly-methylated m^6^A-mRNA rather than single m^6^A motifs can dramatically enhance the phase separation of YTHDF proteins (Gao et al., 2019). On the contrary, G3BP1 and G3BP2, two proteins that contribute to the formation of stress granules that have been suggested to act as m^6^A-readers, have been shown to be repelled by the presence of m^6^A modification in mRNA substrates (Arguello et al., 2017; Edupuganti et al., 2017). These studies underscore m^6^A’s complex role in regulating the fate of cytosolic mRNAs depending on the state of a cell (normal *versus* stress-induced conditions), the number of m^6^A-modified sites (poly-methylated *versus* mono-methylated), and the nature of the RBPs that interact with these modified mRNAs.

Further insights on m^6^A’s role in spatial regulation in neurons derived from studies showing that synapses are preferentially enriched both in m^6^A-modified transcripts and m^6^A regulatory proteins such as FTO, YTHDF1-3 family, and FMRP. Importantly, the presence of these m^6^A-transcripts as well as the m^6^A-regulatory proteins in the synapses was essential for their maintenance and function (Chang et al., 2017; Merkurjev et al., 2018), indicating that the m^6^A-effector proteins might regulate not only the fate of m^6^A-modified targets but also their subcellular distribution.

## Role of m^6^A RNA Methylation in Development and Function of the Nervous System

The widespread distribution of m^6^A modification throughout the mammalian brain in conjunction with functional studies targeting the m^6^A effectors in neural cells highlight m^6^A as an important post-transcriptional regulator of the nervous system (Meyer et al., 2012; Hess et al., 2013; Schwartz et al., 2014; Nainar et al., 2016; Widagdo et al., 2016; Aas et al., 2017; Chang et al., 2017). To date, in the mammalian nervous system, m^6^A regulation has been demonstrated in several physiological functions, including neurodevelopment, learning and memory, synaptic function, as well as injury (Figure 1).

### Neurodevelopment

Accumulating evidence emphasizes m^6^A’s central role in shaping the transcriptome of both neuronal progenitor cells and adult stem cells during neurogenesis in the mammalian brain. In rodents, deletions of writers (*Mettl3*, *Mettl14*), erasers (*Fto*) and readers (*Ythdf2*, *Fmr1*) have profound developmental effects (Fischer et al., 2009; Gao et al., 2010; Yoon et al., 2017; Li et al., 2018). Deletion of *Mettl3* in mouse and human embryonic stem cells leads to a significant decrease in m^6^A levels and severely impairs stem cells transition from self-renewal to differentiation (Batista et al., 2014; Wang Y. et al., 2014; Chen et al., 2015; Geula et al., 2015; Chen J. et al., 2019). Notably, germline deletion of *Mettl3* in mice causes early embryonic lethality (Geula et al., 2015). Conditional knockout of *Mettl14* in the developing mouse nervous system prolongs the cell cycle of radial glia cells and extends cortical neurogenesis into postnatal stages (Yoon et al., 2017). Remarkably, m^6^A-tagged transcripts derived from early embryonic mice forebrains were enriched in genes related to cell cycle and neurogenesis, whereas inhibition of m^6^A attenuated the decay of these transcripts (Yoon et al., 2017). Deletion of *Ythdf2* in mice caused lethality at late embryonic developmental stages, with embryos characterized by delayed cortical neurogenesis (Li et al., 2018). This phenotype is attributed to hindered degradation of m^6^A-tagged genes associated with neural development and differentiation in *Ythdf2* knockout mice, reminiscent of the *Mettl14* knockout defects in cortical neurogenesis (Yoon et al., 2017). Similarly, ablation of FMRP leads to neural differentiation defects due to m^6^A-dependent mRNA nuclear export impairment in *Fmr1* knockout mice (Edens et al., 2019).

Aside from its role in controlling embryonic neurogenesis in the mouse brain, m^6^A was also proposed to affect adult neurogenesis in *Fto* knock-out mice (Li et al., 2018). In particular, loss of FTO was accompanied by a reduced proliferation and differentiation of adult neural stem cells (aNSCs) in the subventricular zone of the dentate gyrus in the hippocampus and this reduction was concomitant with impairments in learning and memory (Li et al., 2018). A more recent study provided further support on the role of m^6^A in adult neurogenesis by demonstrating that depletion of *Mettl3* significantly reduced m^6^A levels in aNSCs, inhibited their proliferation, and affected the morphological maturation of newborn neurons in the adult brain (Chen X. et al., 2019). Importantly, using methylated RNA immunoprecipitation sequencing (Me-RIP-seq) this study uncovered the presence of a crosstalk between m^6^A and histone modifications in aNSCs. Specifically, Me-RIP-seq revealed that m^6^A was present on the transcripts of the histone methyltransferase *Ezh2* (Chen J. et al., 2019). Reduction of m^6^A following *Mettl3* depletion decreased both EZH2 protein expression and the levels of its target H3K27me3, leading to defects in neuronal development, which could be rescued by *Ezh2* overexpression (Chen J. et al., 2019). In support of this crosstalk between m^6^A and histone modifications, a study by Wang Y. et al. (2018) reported that m^6^A can regulate histone modifications directly by destabilizing transcripts encoding histone modifiers, such as the H3K27 acetyltransferase CBP/p300. Therefore, these studies proposed a novel mechanism for gene regulation where m^6^A and epigenetic mechanisms work in unison to safeguard normal embryonic and adult mouse brain neurogenesis.

### Synaptic and Axonal Function

Several studies in neurons have reported that synapses are enriched not only of m^6^A-modified mRNAs but also of m^6^A-effectors (Merkurjev et al., 2018; Yu et al., 2018; Zhuang et al., 2019). Transcriptome-wide m^6^A profiling in the mouse cerebellum and cerebral cortex revealed that m^6^A signatures/peaks were enriched in functions pertaining to transcriptional regulation, axon guidance, synapse assembly and organization (Chang et al., 2017). Notably, m^6^A-sequencing of synaptosomal RNA from healthy adult mouse forebrains revealed that the synaptic m^6^A epitranscriptome is functionally enriched in genes involved in the synthesis and modulation of the “tripartite” synapses, a term referring to presynaptic terminals, postsynaptic terminals, and glia cells (Perea et al., 2009). In the same study, m^6^A-regulatory proteins such as YTHDF1-3, and FTO were localized in extrasomatic regions and in dendrites in adult mouse brain slices and in hippocampal neuronal cultures, respectively. Selective knockdown of readers *Ythdf1* and *Ythdf3* in hippocampal neurons resulted in synaptic dysfunction including immature spine morphology and dampened excitatory synaptic transmission (Merkurjev et al., 2018), suggesting a functional role for m^6^A readers in these synapses.

FTO was shown to be enriched in the axons and most importantly to be locally translated (Yu et al., 2018). Axon-specific inhibition of FTO led to generally increased m^6^A levels and, specifically, m^6^A levels within axonal growth-associated protein-43 (GAP-43) mRNA, whose expression is required for axon elongation (Skene and Willard, 1981). This increase in m^6^A-GAP-43 mRNA suppressed translation of axonal GAP-43 mRNA, which repressed axon elongation in mouse dorsal root ganglia neurons (Yu et al., 2018). In addition to its synaptic function, YTHDF1 was recently reported to regulate axonal guidance in spinal cord through translational control of the axon guidance receptor Robo3.1 (Zhuang et al., 2019). Likewise, other RBPs, such as FMRP (Edupuganti et al., 2017), can act as sequence-context-dependent m^6^A readers and form RNA transport granules, which regulate dendritic localization of RNAs as well as inhibit local synaptic transcript translation (Dictenberg et al., 2008; Kao et al., 2010), highlighting m^6^A’s diverse role in promoting regulation of synaptic function and growth.

### Learning and Memory

Several studies focusing on m^6^A’s role in the nervous system, following perturbations of its machinery, have underlined its involvement in the process of learning and memory. Evidence derived from early genome-wide studies in humans indicates the importance of *FTO* in memory processing (Ho et al., 2010; Benedict et al., 2011). In line with this observation, *Fto* downregulation in specific brain regions can lead to changes in the learning capacity of mice (Widagdo et al., 2016; Walters et al., 2017). In particular, m^6^A levels were shown to be dynamically regulated in the mouse medial prefrontal cortex following post-behavioral training, in fear-conditioned mice, with *Fto* knockdown increasing m^6^A levels and resulting in enhanced consolidation of cued fear memory (Widagdo et al., 2016). Similarly, downregulation of *Fto* in the dorsal hippocampus enhanced contextual fear memory (Walters et al., 2017). Studies on METTL3 have highlighted its direct role in regulating the efficacy of hippocampus-dependent memory consolidation, likely by promoting the translation of neuronal early-responsive genes (Zhang Z. et al., 2018). Interestingly, conditional deletion of *Mettl3* or *Fto* in adult excitatory neurons in mice can increase fear memory and induce transcriptomic alterations in several genes involved in neuronal circuit function, suggesting m^6^A contributes to neuronal circuit regulation (Engel et al., 2018). Deletion of another core writer subunit, *Mettl14*, in the striatum lead to increased neuronal excitability and severely impairs striatal-mediated behaviors (Koranda et al., 2018), highlighting m^6^A’s important role in normal striatal function in adult mice.

m^6^A readers have also been linked to learning and memory related processes (Shi et al., 2019; Wu et al., 2019). YTHDF1-dependent m^6^A protein synthesis of target transcripts, in response to neuronal stimuli, facilitates learning and memory in the adult mouse hippocampus. Mice with genetic deletion of *Ythdf1* exhibit learning and memory defects as well as impaired hippocampal synaptic transmission and long-term potentiation (Shi et al., 2019). Lastly, neural or oligodendroglia-specific knockout of *Prrc2a*, a novel m^6^A reader that controls the specification and myelination of oligodendrocytes, was also reported to be associated with locomotive and cognitive defects in a mouse model (Wu et al., 2019).

### Nervous System Regeneration

Injuries in the adult peripheral nervous system can induce regeneration processes, which are accompanied by *de novo* gene transcription and protein translation of regeneration-associated genes (RAGs). Using a sciatic nerve injury model in adult mice, Weng et al. (2018) reported upregulation of m^6^A-tagged transcripts, encoding many RAGs, to occur up to 3 days post-injury in the dorsal root ganglion neurons (DRGs). Deletion of either *Mettl14* or *Ythdf1* attenuated injury-induced protein translation in DRGs, which consequently hindered the regeneration process in the mouse peripheral nervous system (Weng et al., 2018). Similarly, in the mouse adult central nervous system, regeneration of retinal ganglion cells post-injury was also shown to implicate m^6^A-dependent function (Weng et al., 2018). Recently, m^6^A profiling in the rat cortex following traumatic brain injury (TBI) showed that *Mettl14* and *Fto* were significantly reduced and functional FTO was necessary to repair the neurological damage caused by TBI (Yu et al., 2020). These early studies suggest that m^6^A might exert a broader role in response to pathological stimuli in the adult mammalian nervous system.

## RNA m^6^A Methylation in Neurological Diseases

Considering the widespread effects of m^6^A modification on gene expression, it is not surprising that aberrant methylation could underlie the disruption of RNA processing events that are increasingly implicated in neurological diseases (Nussbacher et al., 2019). Initial indication that m^6^A modification may be involved in the etiology of neurological diseases comes from genome-wide association studies in which several variants in genes encoding for components of the m^6^A machinery were associated with the risk of developing neurological disorders, such as intellectual disability, depression, schizophrenia, and Alzheimer’s disease (Engel and Chen, 2018). In this section, we will review the link between altered m^6^A deposition on mRNA and susceptibility to neurological diseases, broadly classified as neurodevelopmental, mood, and neurodegenerative disorders.

### m^6^A Dysfunctions Leading to Neurodevelopmental Disorders

Neurodevelopmental disorders (NDDs) are a group of disabilities which typically manifest early in development. They are characterized by brain dysfunction which can lead to neuropsychiatric problems or impairments in cognition, language, behavior, memory, motor skills or other neurological functions (Reiss, 2009). While NDD symptoms and behaviors often evolve as a child grows older, some disabilities such as attention deficit hyperactivity disorder (ADHD) and autism spectrum disorder can be lifelong conditions (Thapar et al., 2017). Treatment of these disorders can be complex and it often involves a combination of behavioral therapy and medications.

Initial indication that altered RNA methylation could be linked to the etiology of NDDs stems from the observation that a loss-of-function mutation in the *FTO* gene was linked with structural and functional brain abnormalities, as well as severe motor delay (Boissel et al., 2009). ADHD is the most common NDD observed in childhood with symptoms that include inattention, hyperactivity, and impulsivity (Doernberg and Hollander, 2016). Interestingly, a duplication of the *FTO* gene results in intellectual disability and ADHD (van den Berg et al., 2010). Velders et al. (2012) provided the first indication that the *FTO* allele at rs9939609 is linked with decreased risk for symptoms of ADHD and fewer challenges with emotional control in young children. Choudhry et al. (2013) corroborated these observations implicating FTO in the modulation of ADHD phenotype with a more obvious effect in those children who were not exposed to smoke during gestation.

Notably, m^6^A is enriched at synapses (Shafik et al., 2020) and aberrant translation at synapses has long been associated with autism and other intellectual disorders such as the Fragile X syndrome (FXR). FXR is the most common form of inherited intellectual disability caused by the expansion of trinucleotide repeats in the 5′ UTR of the *FMR1* gene, resulting in reduced levels of the encoded protein FMRP (Santoro et al., 2012). Suggestive of a role for m^6^A in these forms of intellectual disabilities, mutations in the *YTHDC2* gene have been implicated as a risk factor for autism spectrum disorder (Liu X. et al., 2016). In addition, in line with a likely association between m^6^A regulation and intellectual disability, a study found that m^6^A is present in many mRNAs that are known synaptic targets of FMRP (Chang et al., 2017). Moreover, FMRP has been shown to stimulate nuclear export during neural differentiation by binding to m^6^A-modified sites on several mRNA (Edens et al., 2019; Hsu et al., 2019).

Interestingly, Yoon et al. (2017) also reported that several transcripts associated with schizophrenia−a mental disorder with roots in early brain development and characterized by disconnection with the reality (Stepnicki et al., 2018)−are m^6^A-modified in human, but not in mouse cultures of neuronal progenitor cells, suggesting a specific role for m^6^A in this disease (Yoon et al., 2017). In line with this observation, polymorphisms in the *ZC3H13* gene have also been linked with schizophrenia (Oldmeadow et al., 2014).

### m^6^A Dysfunctions Leading to Mood Disorders

Mood disorders are a group of emotional disturbances characterized by prolonged periods of excessive happiness, excessive sadness, or both. Mood disorders can cause behavioral changes that can impact pragmatic and executive functioning (Sekhon and Gupta, 2020). Two of the most common mood disorders are major depression disorder (MDD) and bipolar disorder. Psychotherapy, antidepressants, and support can help treat mood disorders. In particular, there is evidence of an impairment of glucocorticoid receptor-mediated negative feedback on the hypothalamic-pituitary-adrenal (HPA) axis resulting in a glucocorticoid resistance in MDD (Holsboer, 2000). Importantly, successful treatment with antidepressant can lead to resolution of glucocorticoid resistance (Heuser et al., 1996).

A survey of Chinese Han people has shown an association between specific *ALKBH5* polymorphisms and MDD, raising the possibility that *ALKBH5* is involved in conferring a higher risk of this mood disorder (Du et al., 2016). In addition, altered levels of m^6^A were observed in depressed patients after glucocorticoid stimulation (Engel et al., 2018; Spychala and Ruther, 2019). Specifically, Spychala and Ruther (2019) reported that *Fto* loss-of-function results in increased stress parameters such as corticosterone in the blood, suggesting hypersensitivity of the HPA axis. A processing defect of brain-derived neurotrophic factor (BDNF)−a neurotrophic factor involved in the pathophysiology of MDD (Lee and Kim, 2010)−was found in the hippocampus of these *Fto* knockout mice, which presented with deficits in working memory and increased anxiety (Spychala and Ruther, 2019). Engel et al. (2018) further elucidated the role of m^6^A in the context of stress response in adult mice. By examining mice with *Mettl3* and *Fto* depletion in excitatory neurons they found alterations on m^6^A profiles with concomitant changes in transcriptome regulation, behavior, and electrophysiological properties (Engel et al., 2018). Importantly, they also reported that m^6^A regulation in blood could be used as a peripheral proxy of the brain’s m^6^A responses that are impaired in MDD patients (Engel et al., 2018).

### m^6^A Dysfunctions Leading to Neurodegenerative Disorders

Neurodegenerative disorders are a heterogeneous group of illnesses that are characterized by the progressive demise of neurons in different regions of the nervous system. As neurons deteriorate, neurological signs and symptoms characteristic of each of these disorders arise and progressively worsen. In some cases, patients may lose the ability to walk independently, think clearly, or perform their daily functions. Ultimately, many of these diseases are fatal (Dugger and Dickson, 2017). Neurodegenerative disorders affect millions of people worldwide, with Alzheimer’s disease (AD) and Parkinson’s disease (PD) being the most common. Although treatments may help relieve some of the physical or neurological symptoms associated with neurodegenerative disorders, currently there is no cure.

In older adults, AD is the most common form of dementia that is caused by damage in several areas of the brain, which include the hippocampus and the frontal lobe. As a result, patients have difficulties to form new memories and eventually have problems with intellect, judgment, and behavior (Weller and Budson, 2018). Several lines of evidence link genetic variations in the *FTO* gene with a greater risk of Alzheimer’s disease (AD) and related dementias. First, in a large magnetic resonance imaging (MRI) study of healthy elderly subjects, Ho et al. (2010) reported substantial brain tissue deficits in carriers of the *FTO* rs9939609 risk allele. Subjects carrying one copy or more of the *FTO* rs9939609 allele presented with brain atrophy in the frontal lobe, an area important for language and experience-dependent functions of the brain (Ho et al., 2010). Second, studying a cohort of elderly subjects who had no clinically overt cognitive dysfunction, Benedict et al. (2011) found that only obese and overweight but not normal weight *FTO* risk allele carriers showed reduced language performance relative to non-carriers. However, general cognitive performance remained unaffected in the obese and overweight subjects, suggesting that the *FTO* gene influences mainly frontal lobe-dependent brain functions (Benedict et al., 2011). Third, in a longitudinal cohort study, Keller et al. (2011) found an association between the *FTO* risk allele and the risk of developing AD and dementia. These findings were confirmed and extended in another study that reported a link between certain polymorphisms in intron 1, exon 2, or intron 2 of the *FTO* gene and AD in Caucasians of European ancestry as well as in Caribbean Hispanics (Reitz et al., 2012). In addition, in two independent datasets they found significantly lower levels of *FTO* in AD cases compared to controls and a positive association between the polymorphism residing in intron 2 and *FTO* expression levels (Reitz et al., 2012). Finally, a recent study reported that the expression of *Mettl3* increased in AD mice relative to control mice, while the expression of *Fto* decreased, which was in line with the increased methylation observed in the AD group (Han et al., 2020). Genes related to synaptic function were the most abundant group of transcripts among those with changes in m^6^A RNA methylation levels, providing further support for a role of altered m^6^A regulation in the etiology of AD and related dementias (Han et al., 2020).

Parkinson’s Disease is a progressive disorder that affects predominately dopamine-producing neurons in the area of the brain which controls movement, called the substantia nigra. Normally, dopamine functions in a delicate balance with other neurotransmitters to help coordinate the neural circuit in the basal ganglia that controls movement. Without enough dopamine, this balance is altered, resulting over the years in slowness of movement, lack of spontaneous motility, resting tremor and rigidity (Przedborski, 2017). Because glutamate receptors (NMDARs) modulate synaptic transmission throughout the basal ganglia, and pharmacological manipulation of these receptors can correct aberrant neurotransmission such as that observed in the parkinsonian brain, NMDAR have been proposed as therapeutic targets for treating PD (Johnson et al., 2009). Evidence is also emerging in support of an involvement of FTO and m^6^A aberrant regulation with the occurrence of PD. For instance, electrophysiological analysis of both conventional and conditional *Fto* deficient mice showed an impaired dopamine type 2 (DR2) and 3 (DR3) receptor response characterized by a reduced cocaine-mediated suppression of firing rate (Hess et al., 2013). This view is further substantiated by the finding that selective deletion of *Fto* in dopaminergic neurons resulted in an increased sensitivity toward cocaine-induced locomotor activity (Hess et al., 2013). While these findings involve FTO and m^6^A in the control of D2R- and D3R- dependent signaling, whether humans carrying polymorphisms in the *FTO* gene have aberrant D2R- and D3R-dependent behaviors is still unknown. In a recent study using PC12 cells and rats treated with 6-OHDA to model the disease, Chen X. et al. (2019) more closely investigated the role of m^6^A modification in the pathogenesis of PD. They found that m^6^A modification is downregulated in PC12 cells and in the substantia nigra of rats after treatment with 6-OHDA. They also reported an increase in the expression level of glutamate receptor subunit 1 (Nmdar1), which could be a consequence of reduced m^6^A modification. Consistent with this hypothesis, they reported that *Fto* deficiency in PC12 cells caused a reduction in Nmdar1 levels and prevented cell death. Altogether, these observations support a critical role for m^6^A modification in the pathogenesis of PD (Chen X. et al., 2019).

Amyotrophic Lateral Sclerosis (ALS) is a fatal neurodegenerative disorder which combines clinical evidence of both upper and lower motor neuron degeneration with patients resulting in dysfunction of the somatic muscles (Rowland et al., 2010; Saberi et al., 2015; Grad et al., 2017). About 90% of all cases of ALS are sporadic (sALS), while the remaining ∼10% is familial (fALS). Familial cases are inherited through an autosomal dominant pattern due to mutations in several genes (Alsultan et al., 2016; Grad et al., 2017). Usually, ALS is a disease of mid and late life with only 10% beginning before the age of 40 (Rowland et al., 2010). However, the onset is highly variable among patient and often earlier in fALS compared to sALS cases, despite the fact that the two forms of the disease are phenotypically indistinguishable (Swinnen and Robberecht, 2014; Grad et al., 2017). The rate of progression is also highly variable (Swinnen and Robberecht, 2014) even though the mean survival from the time of diagnosis is approximately 4 years with less than 20% surviving more than 5 years (Rowland et al., 2010). Evidence of m^6^A involvement in the pathogenesis of ALS is also emerging. In addition to its wide expression in brain, FTO has been shown to be highly expressed in lower motor neurons (Mitropoulos et al., 2017). Whole-genome sequencing analysis of Greek patients with sporadic ALS revealed a positive association between *FTO* gene variants and the disease (Mitropoulos et al., 2017). Moreover, a targeted gene sequencing of RBPs in ALS patients identified rare deleterious polymorphisms at a significantly higher rate than control in the *RBM15* gene and in its paralog *RMB15B* (Cooper-Knock et al., 2017). In addition to being part of the m^6^A methyltransferase complex, *Nito*, the *Drosophila* homolog of human *RBM15*, was reported to regulate axon outgrowth, branching and to control synaptogenesis via the activity of the bursicon-expressing neurons (Gu et al., 2017). One further observation that encourages investigation on the potential involvement of m^6^A in motor neuron degeneration is that mutations in the gene coding for the m^6^A reader hnRNPA2-B1 can cause ALS (Kim et al., 2013).

Spinocerebellar ataxias (SCAs) are a group of hereditary disorders that are characterized by loss of balance and coordination accompanied by slurred speech. SCAs are due to degenerative changes in Purkinje neurons in the cerebellum, with resulting cerebellar atrophy. In some cases, other parts of the nervous system, such as the spinal cord, basal ganglia and pontine nuclei, can be affected in the disease (Klockgether et al., 2019). A link between m^6^A and SCA is suggested by studies reporting an essential role for m^6^A in the regulation of postnatal cerebellar development (Ma et al., 2018; Wang C. X. et al., 2018). In particular, one study reported that selective *Mettl3* deletion in the nervous system leads to a severe reduction of m^6^A together with prominent developmental defects in the cerebellum of these mice that present typical features of cerebellar ataxia (Wang C. X. et al., 2018). Wang C. X. et al. (2018) further reported that Mettl3 and m^6^A participate to cerebellar development by modulating RNA stability of development- and apoptosis-associated genes and by controlling the alternative splicing of synapse-associated transcripts. Ma et al. (2018) substantiated these observations showing that aberrant expression of METTL3 and ALKBH5 resulted in imbalanced m^6^A, which in turn led to aberrant cerebellar development (Ma et al., 2018). Interestingly, in line with a potential involvement of m^6^A in the pathogenesis of SCA, reduced level of *Ythdc1* was found to enhance neurodegeneration in a *Drosophila* model of SCA-1 (Fernandez-Funez et al., 2000).

## Conclusion

The nervous system requires multiple levels of regulation to establish proper circuit assembly and function. RNA modifications (epitranscriptomics) are a new addition to the complex world of post-transcriptional gene regulation that can control the fate and function of both coding and non-coding RNAs. RNA modifications are preferentially enriched in neurons and are increasingly becoming critical determinants of neuronal function. The fast turnover and pervasiveness of RNA molecules, combined with the dynamic nature of various RNA modifications, allow epitranscriptomics to become a primary regulatory mechanism of cellular response to environmental cues. Based on indications that have linked aberrant m^6^A methylation with brain disorders, this specific RNA modification is emerging as an important regulator of neural cell fate decisions and plasticity. Despite these discoveries, our mechanistic understanding of m^6^A signaling in neuronal physiology and its contribution to pathology are still largely unexplored.

Due to the abundance of m^6^A-modified mRNAs in neurons, one of the major challenges is to identify the RNAs that are targeted by m^6^A in individual cells, characterize the effect of the modification on gene expression, and then link the m^6^A-dependent effect with normal and pathological behavior *in vivo*. The availability of conditional knockout mice for most of the m^6^A writers, erasers and readers is facilitating our understanding of the consequences of manipulation of m^6^A at the cellular and behavioral levels. However, mutation of a single m^6^A site, rather than manipulation of common m^6^A enzymes, is required to functionally uncover the consequences of site-specific methylation. Unfortunately, the lack of tools for a specific spatiotemporal manipulation of single m^6^A sites *in vivo* still limits the causal investigation of the functional outcomes of covalent RNA modulation. Several clustered regularly interspaced short palindromic repeats (CRISPR)-based approaches that in addition to targeting mRNA directly are also carrying RNA-modifying enzymes to specific targets may soon become a valuable toolkit toward this goal (Abudayyeh et al., 2016; East-Seletsky et al., 2016; Liu L. et al., 2017).

Along the same line, reliable quantification of m^6^A dynamics in different brain cells will be crucial to identify the downstream consequences of specific methylation events. Currently, methods to precisely discriminate “regulated” from “constitutive” m^6^A sites require large quantities of material or are limited to global or low-throughput gene- and site-specific determination. Recent development of new methods to enable analysis of m^6^A stoichiometry, such as RNA digestion via m^6^A sensitive RNase (MAZTER-seq) (Garcia-Campos et al., 2019) and Nanopore (Garalde et al., 2018), may overcome these limitations and will allow to determine to which extent the RNA methylation can be dynamically regulated in order to control gene expression. For this purpose, it will also be crucial to integrate findings of mRNA methylation profiles with analysis of RNA levels, alternative polyadenylation, alternative splicing, localization, and translation efficiency. By linking m^6^A-mediated effects on gene expression to specific neurological defects, these investigations have the potential not only to advance our mechanistic understanding of brain disorders but also to discover and develop new therapeutics based on rational manipulation of specific epitranscriptomic processes.

## Author Contributions

GD and FL researched the data for the review and wrote, reviewed, and edited the manuscript before submission. Both authors contributed to the article and approved the submitted version.

## Conflict of Interest

The authors declare that the research was conducted in the absence of any commercial or financial relationships that could be construed as a potential conflict of interest.
